# Motorist’s Vestibular Disorientation Syndrome (MVDS)—Proposed Diagnostic Criteria

**DOI:** 10.3390/jpm13050732

**Published:** 2023-04-26

**Authors:** Vishal Pawar, Hanaan Ashraf, Srinivas Dorsala, Preethy Mary, Nazrin Hameed, Divya Nair H, Sweta Prakash Adatia, Leya Raj, V. R. Ananthu, M. Shouka

**Affiliations:** 1 Neurology Department, Aster Gardens Specialty Clinic, Building 10, Zen Cluster, Street 1, Discovery Gardens, Dubai P.O. Box 8703, United Arab Emirates; 2 Al Rafa Polyclinic-International City, Internal Medicine Department, Aster DM Healthcare, Dubai P.O. Box 8703, United Arab Emirates; 3 Ear, Nose and Throat (ENT) Department, Jawaharlal Nehru Medical College (JNMC), Belagavi 500010, Karnataka, India; 4 Medical Trust Hospital, Department of Otolaryngology, Cochin 682016, Kerala, India; 5 Indian Institute of Public Health Gandhinagar, Gandhinagar 382042, Gujarat, India; 6 Department of Neurology, RAK Hospital, Ras-Al-Khaimah P.O. Box 11393, United Arab Emirates; 7 Public Health Consultant, Karipuram 673121, Kerala, India; 8 Al Sharq Hospital Fujairah, ENT Department, Al Sharq Healthcare, Fujairah P.O. Box 8505, United Arab Emirates; 9 Audiologist, Neurology Department, Garden’s Specialty Clinic, Aster DM Healthcare, Dubai P.O. Box 8704, United Arab Emirates

**Keywords:** spatial orientation, dizziness, disorientation, driving, vehicles

## Abstract

Motorist’s vestibular disorientation syndrome (MVDS) is a disorder in which patients experience dizziness while driving. MVDS is under-reported in the literature, and in clinical practice, it often goes unrecognized. We identified clinical characteristics of patients with MVDS using data from 24 patients who faced difficulties while driving and were diagnosed with MVDS. Their symptoms, duration of illness, precipitating factors, co-morbidities, history of other neuro-otological disorders, severity of symptoms, and associated anxiety and depression were reviewed. Ocular motor movements were recorded using video-nystagmography. Patients with vestibular disorders that can cause similar symptoms while driving were excluded. The mean age of the patients was 45.7 ± 8.7 years, and most were professional drivers (90.5%). The duration of the illness ranged from eight days to ten years. Most patients presented with disorientation (79.2%) exclusively while driving. The most common triggers for symptoms were higher speeds, i.e., >80 km/h (66.7%), multi-lane roads (58.3%), bends and turns (50%), and looking at other vehicles or signals while driving (41.7%). A history of migraines was reported in 62.5% of the patients, and motion sickness was reported in 50% of the patients. Anxiety was reported in 34.3% of patients, and 15.7% had depression. The video-nystagmography did not show any specific abnormalities. Patients responded to drugs used in prophylactic treatments for migraines such as Amitriptyline, Venlafaxine, Bisoprolol, and Magnesium, and to Pregabalin and Gabapentin. Based on these findings, a classification system and a diagnostic criterion for MVDS were proposed.

## 1. Introduction

Driving a vehicle in the dynamic environment of modern highways is a learned skill that reflects spatial cognitive ability [1,2] and involves the coordination of all four limbs. The various parts of the neuraxis involved in driving include higher cognitive functions such as visuospatial orientation, memory, and the visual cortex. Cranial nerves, including the optic nerves for vision, ocular motor nerves for extra-ocular movements, vestibulocochlear nerves for vestibular and auditory cues, and spinal accessory nerves for neck movements are also involved. The motor system (voluntary control of upper limbs, trunk, and lower limbs), sensory system (proprioception, touch, and vibration sense), and the cerebellar system are also necessary for the act of driving, and the extra-pyramidal system is required for maintaining the tone of muscles [1,3].

The vestibular labyrinth provides information about linear and angular acceleration of the head to central areas of the brain such as the brainstem, cerebellum, and vestibular cortex. It detects and measures movements in response to self-motion (active motion) and externally induced forces (passive motion) and is a crucial component in regulating gaze stability and postural reflexes [4]. The vestibular system is a critical organ for balance and spatial navigation, and its dysfunction can cause balance disorders and spatial disorientation [5].

The vestibular system plays a vital role in spatial navigation and head orientation by judging the speed of head movement. The visual system provides input regarding the differential movement of the visual field (optic flow) during driving, which is important due to the paucity of visual cues during driving [2]. Asymmetrical optic flow can create challenges while driving, for example when a big truck obstructs the optic flow from one side of an open highway.

The vestibular, visual, and proprioceptive systems coordinate with each other to adjust outgoing motor responses contributing to balance, posture, and stabilization of gaze. An impairment in balance can arise due to central or peripheral components of the vestibular system that project an illusion of self-motion like dizziness or vertigo and disorientation in space [5].

**Figure 1:**
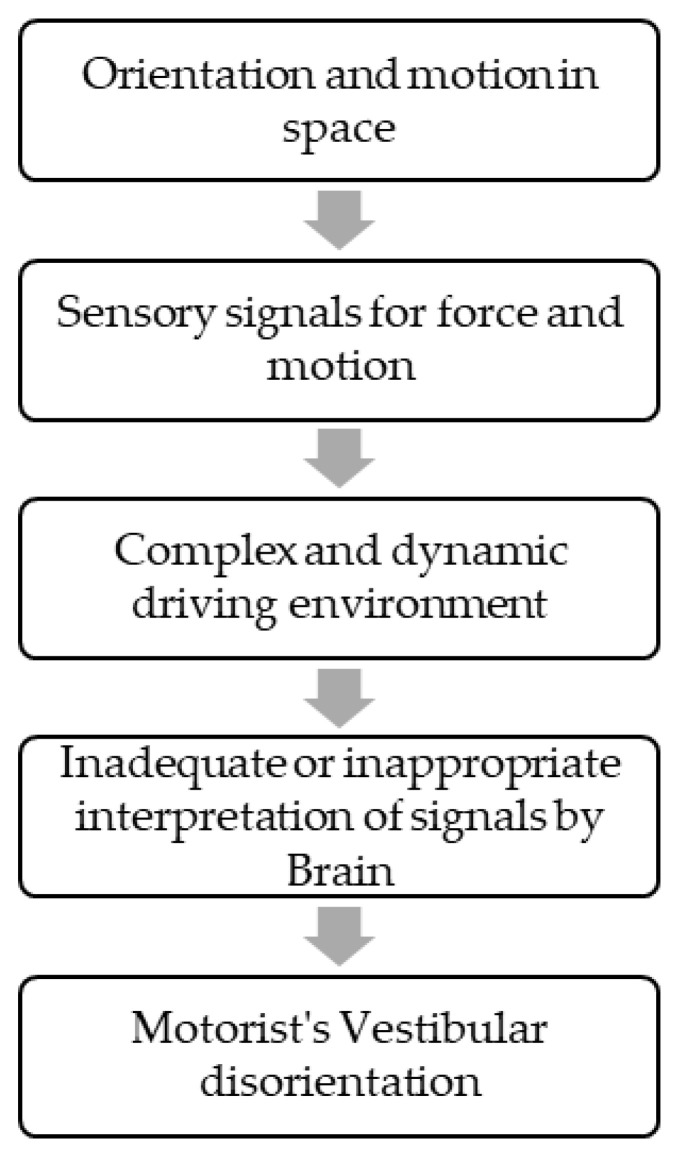
Manifestation of Symptoms.

Vestibular symptoms as a passenger, i.e., motion sickness, are a well-known entity. Vestibular symptoms can also happen while driving in some vestibular disorders such as vestibular migraines, Meniere’s disease, etc. [5]. In 1985, Page et al. described patients who had episodes of disorientation exclusively while driving a car. This state of spatial disorientation was attributed to inadequate or misinterpreted sensory signals by central brain areas responsible for orientation and motion in space (Figure 1).

The term motorist’s vestibular disorientation syndrome (MVDS) was coined to describe these symptoms [6,7]. Similar symptoms are commonly reported in aviators [8]. A distorted sense of motion perception while driving can make drivers feel like they are uncontrollably veering off the road or rolling over corners, which can affect their attention and driving [8,9]. MVDS patients are also susceptible to visual vertigo, motion sickness, anxiety, and phobia [7]. This experience of disorientation is frequently attributed to vestibular dysfunction, along with dysfunctional canalicular and otolithic systems that worsen while driving [6]. Symptoms while driving are commonly reported in other vestibular disorders such as vestibular migraine, persistent postural perceptual dizziness (PPPD), Meniere’s disease, benign paroxysmal positioning vertigo (BPPV), chronic vestibulopathy, and in patients with postoperative acoustic neuroma resection or vestibular nerve section [9,10]. These symptoms can also appear without driving in these disorders.

In clinical practice, MVDS, though less common, is not unfamiliar. However, since its first description in 1985, literature exploring the syndrome’s characteristics has seldom been reported, making it an understudied and underreported phenomenon. As there is a rising concern about the possibility of accidents endangering the safety of patients with MVDS while driving, there is a need to understand the patient characteristics of MVDS to aid in early diagnosis and treatment.

This study provides insights into clinical features and video-nystagmography (VNG) findings in MVDS patients. VNG findings help in making a clinical diagnosis by recording eye movements in graphical as well as video recording formats, and aid in differentiating between a central or peripheral vestibular lesion [11,12]. We also suggest a classification system, diagnostic criteria, and treatment for MVDS.

## 2. Materials and Methods

A total of 24 patients presenting with signs and symptoms of MVDS, who approached the vertigo clinic in Dubai, United Arab Emirates, and Cochin, India, between January 2018 and September 2021 were enrolled in the study.

Patients presenting with the symptoms listed below were included in the study:

- Patients presenting with one or more symptoms of dizziness while driving, including:
  - Disorientation
  - False perception of the vehicle turning to one side
  - Sweating, Palpitations, cold extremities
  - Stiffness of body
- Symptoms present exclusively while driving and aggravated during the following situations:
  - Higher speed (>80 km/h)
  - Bends and turns
  - Multi-lanes
  - Upward or downward slopes
  - Looking down from bridges
  - Overtaking
  - Looking at other vehicles while driving
  - Closed tunnels
- Symptoms leading to significant distress or functional impairment while driving
- Symptoms that were not better attributed to another disease or disorder

Patients having symptoms of visual vertigo de novo or due to vestibular migraine or PPPD without driving were excluded from the study. Patients manifesting other vestibular diseases such as Meniere’s disease, BPPV, etc., and those who experienced dizziness exclusively as a passenger (motion sickness) were excluded from the study.

The medical records of 24 patients diagnosed with MVDS were reviewed in detail. Their demographic data, including age, sex, occupation, symptoms, duration of symptoms, precipitating factors, co-morbidities, history of neuro-otological disorders, and psychiatric illnesses were recorded.

The symptom severity was assessed using a 25-item self-reported Dizziness Handicap Inventory (DHI) [13]. Patients were evaluated for their visual, postural, and movement scores using the NIIGATA PPPD scale [14]. Patients also underwent assessments for anxiety and depression using the Generalized Anxiety Disorder-7 (GAD-7) [15] and Patient Health Questionnaire-9 (PHQ-9) [16], respectively.

Ocular motor movements were recorded using video-nystagmography goggles (Balance Eye Binocular VNG system Cyclops MedTech Pvt. Ltd., Bengaluru, India). The tracing obtained were of horizontal and vertical eye movements with simultaneous recording for eye video. Brain MRIs and other vestibular tests were performed as needed.

Patients who met the inclusion criteria and were diagnosed with MVDS and treated accordingly. A variety of drugs based on co-morbidity were used, such as Amitriptyline, Bisoprolol, Magnesium, Venlafaxine, Desvenlafaxine, Gabapentin, and Pregabalin. The response to each treatment modality was recorded during follow-up visits. Patients underwent assessments after one month of the treatment and during subsequent follow-ups using the Dizziness Handicap Inventory. The natural history of the symptoms was also recorded.

Data were analyzed using SPSS Statistics for Windows, Version 20.0 (IBM SPSS Statistics for Windows, Version 23.0. Armonk, NY, USA: IBM Corp), and Microsoft Excel 2016. Categorical data including demographics, clinical characteristics, and video-oculography findings were summarized as frequency and percentage. In contrast, continuous variables such as smooth pursuit and optokinetic asymmetry values were summarized as mean, median, and quartiles.

## 3. Results

A total of thirty patients who reported dizziness while driving were screened for MVDS. However, three patients were diagnosed with vestibular migraine, two with PPPD, and one with Meniere’s disease. These patients also had symptoms without driving and were therefore excluded from the study. Among the remaining 24 MVDS patients enrolled in the study, 21 were recruited from the UAE and three from India. The mean age of the patients was 45.7 ± 8.7 years. Most of the patients were professional drivers (90.5%) with a minimum driving experience of 5 years.

Table 1 summarizes the clinical characteristics of the patients, including symptoms associated with MVDS, duration of symptoms, and co-morbidities. The duration of illness was between 8 days to 10 years (mean 2.4 years). We observed that most patients had a history of migraine (62.5%). Among the symptoms of MVDS, most patients experienced dizziness or disorientation (79.2%), followed by a sense of losing control over the vehicle (54.2%) and palpitations (41.7%).

Among the patients, 70.8% reported experiencing symptoms until they pulled over, and in 45.8% of the patients, the symptoms lasted for 1 min to 20 min. Motion sickness as a passenger was reported in 50% of the patients. The severity of dizziness reported by patients on a scale of 1-10 had a mean score of 6.1 ± 2. When asked about the trigger of these symptoms, most patients reported higher speeds (>80 km/h) (66.7%), multi-lane roads (58.3%), bends and turns (50%), looking at other vehicles or signals while driving (41.7%), and other factors. Out of the 24 patients, nine (37.5%) felt that the vehicle veered to the right side, eight (33.3%) felt it veered to the left, and seven (29.2%) did not feel any veering. The majority (62.5%) of the patients did not report any diurnal variation in symptoms.

The PPPD score based on the Niigata questionnaire ranged from 0 to 27, with a median (Q1, Q3) score of 2.5 (0, 7.75). None of the patients had a high enough visual score (>9/24) to qualify for visual vertigo or PPPD. While most patients did not have anxiety (65.2%), 17.4% had severe anxiety when assessed using the GAD-7. Similarly, the majority did not show any depressive symptoms (83.3%), as evaluated by the PHQ-9.

Video-nystagmography (VNG) or video-oculography recordings of 17 patients are summarized in Table 2. VNG recordings of the remaining seven patients were not performed, as some did not consent to the test and others were lost to follow-up.

Normal horizontal saccades were observed among all the patients, but 11.8% had hypometric vertical saccades. The horizontal and vertical smooth pursuit was normal in 76.5% and 5.9% of patients, respectively. Up-beating spontaneous nystagmus was observed in three patients (17.6%), and three (17.6%) had nystagmus induced by head shaking and hyperventilation.

During gaze testing with fixation, none of the patients had any abnormalities. During gaze testing without fixation, 20%, 13.3%, 12.5%, 12.5%, and 13.3% patients had nystagmus in the centre, left, right, up, and down gaze, respectively (Table 3).

We also recorded horizontal smooth pursuit (HSP) and optokinetic (OK) asymmetry of the patients. Those who felt the vehicle veering to the right had a higher mean (SD) HSP asymmetry of −4.6 (12.9) °/s and a horizontal OK asymmetry of −4.1 (6) °/s compared to those who felt the vehicle turning to the left.

The mean difference (SD) in the DHI score before and after treatment was 35.5 (20.7) in four patients after treatment. Post-treatment DHI scores could not be recorded in the remaining patients who underwent treatment, as some of them were lost to follow-up and some did not respond to the questionnaire. However, we have recorded the subjective improvements among patients depending on the treatment provided (Table 4).

## 4. Discussion

The present study was conducted to understand the clinical characteristics of patients presenting with motorist’s vestibular disorientation syndrome. Although the syndrome and its characteristics were identified in 1985 by Page and Gresty [6], it has been understudied and under-reported in the literature. A study from the USA classified participants in the National Health and Nutrition Examination Surveys (NHANES) 2001–2004 based on driving difficulty and found that patients with vestibular dysfunction had a higher prevalence (74.9%) of experiencing driving difficulty. According to NHANES, participants with vestibular dysfunction had an increased risk of suffering from falls. Moreover, those having both vestibular dysfunction and driving impairment were 13 times more likely to fall [17]. These findings highlight the importance of identifying the characteristics of MVDS to prevent falls and accidents.

To the best of our knowledge, this is the second study after Page and Gresty [6] to report on patient characteristics diagnosed with MVDS. In this study, we assessed 24 patients manifesting difficulties while driving for symptoms, co-morbidities, triggers, duration of episodes, video-nystagmography findings, and the presence of anxiety, depressive disorders, and dizziness severity assessed by the DHI.

MVDS patients in the present study were predominantly driving professionals who had the illness for a duration of 8 days to 10 years. Other studies have attributed this wide range of illness duration to a delay in diagnosis, which may be due to a lack of awareness of the disorder, motorists assuming it was an issue related to the car, and not knowing which doctor to consult [7,9]. Patients in our study had multiple consultations with different specialties before receiving a diagnosis of MVDS. Some patients retook driving classes, and others consulted an ophthalmologist. During the latter part of the study, a patient was diagnosed with MVDS after experiencing symptoms for only eight days due to increased awareness of the condition. Patients suffering from MVDS in the present study used the following coping strategies to manage driving-related dizziness [7]:

- Stopping or reducing the frequency of driving
- Avoiding long journeys
- Avoiding multi-lane highways
- Avoiding driving on bridges
- Reducing overtaking
- Reducing the speed well in advance of traffic signals
- Driving at low speeds (less than 80 km/h) on single-lane roads
- Taking frequent breaks on long journeys
- Keeping water/juice ready during the journey

MVDS occurs as a result of inadequate or misinterpreted sensory signals related to orientation and motion in the space of the complex and dynamic driving environment [8] Most of the patients in this study had a current or past history of migraines, but episodes of vestibular symptoms happened exclusively while driving. Vestibular involvement is well-documented in patients with vestibular migraines [18,19], although none of the patients satisfied the criteria for vestibular migraines in our study [15]. It is therefore unclear whether these patients had vestibular migraines. Surprisingly, patients in the present study improved when treated with drugs used for migraine prophylaxis. The association between migraines and MVDS needs to be explored in further studies.

Dizziness or disorientation was the most commonly reported symptom among patients in the present study [5]. Visual vertigo can occur with exposure to moving stimuli, causing the feeling of dizziness [16,20]. Patients with symptoms of visual vertigo in situations other than driving were excluded from our study. Similarly, motion sickness is not a typical feature of MVDS, but a moving environment can induce dizziness and nausea related to motion sickness [7]. In the present study, half of the patients reported motion sickness, even as passengers. This higher incidence of co-morbid migraines can explain the co-existence and susceptibility to motion sickness in MVDS patients [21]. A tonic imbalance in roll or impaired smooth pursuit of eye movements have been reported in central vestibular diseases such as vertigo syndromes and basilar/vestibular migraines [22]. In the present study, although there was an increase in horizontal smooth pursuit when patients felt the vehicle veering to the right, there was no data from normal subjects to substantiate this finding as an impairment in MVDS patients.

Golding and Gresty et al. reported anxiety as a triggering factor for disorientation while driving [8]. Psychiatric co-morbidities were found in 16-17% of patients in the present study. Severe anxiety on the GAD-7 scale could lead to reciprocal functional interactions between anxiety and vestibular systems. Psychiatric co-morbidities are also highly prevalent among patients with Meniere’s disease, vestibular migraines, and also in PPPD [23,24,25].

Previous case studies on MVDS patients [6,7,17] did not explore video-nystagmographic or video oculography findings, which record real-time eye movements including nystagmus [26]. In the present study, video-nystagmography did not show any specific abnormalities. Most patients in the current study had normal horizontal smooth pursuit. However, the vertical smooth pursuit was abnormal in all patients except one. Page et al. reported smooth pursuit impairment in two cases. They also observed spontaneous nystagmus in five out of six cases [6], which was present only in three out of 17 patients without fixation in our study. A few patients in our study exhibited vertical and horizontal smooth pursuit asymmetry, optokinetic asymmetry with symptoms on the slope, a feeling of veering of the vehicle, and nystagmus on gaze testing without fixation. However, these findings did not reach statistical significance. Page et al. observed no asymmetry in response to optokinetic stimuli [6].

In the present study, among the patients who were present on follow-up, the DHI score of 24 before treatment with Amitriptyline, Bisoprolol, and Magnesium, was reduced to 8 in one case. In another case, a score of 60 decreased gradually to 30, then 20, and finally to 2 when treated with Amitriptyline followed by Bisoprolol and Magnesium, and eventually replacing Amitriptyline with Desvenlafaxine. Amitriptyline, in another case, also reduced DHI scores from 52 to 4. One of the patients exhibiting vascular loops on an MRI brain scan was treated with Gabapentin, and their DHI score reduced from 36 to 16. However, we could not make a decisive conclusion due to the limited number of patients that were available on follow-up. We found that a few patients showed 80% improvement when treated with migraine medications such as Amitriptyline, Bisoprolol, Magnesium, or Desvenlafaxine. This link between MVDS and migraines needs to be explored in future studies [8]. Amitriptyline, a tricyclic antidepressant, is commonly used for prophylaxis of vestibular migraine [27].

Considered initially as a subset of phobic postural vertigo, MVDS can be regarded as a task-specific functional neuro-vestibular disorder [20]. Patients with MVDS do not meet the diagnostic criteria of PPPD [9,20]. Hence, until there is an established physiological mechanism to identify MVDS, we propose the following possible classification for motorist’s vestibular disorientation (MVD).

- Primary MVD, i.e., MVDS: symptoms are exclusively experienced while driving, and the patient has no other vestibular disorder to explain the symptoms.
- Secondary MVD: the patient has an underlying vestibular disorder explaining the symptoms, and the symptoms are not exclusive to driving.
  ○ Secondary to visual vertigo in vestibular migraine
  ○ Secondary to visual vertigo in PPPD
  ○ Secondary to other vestibular disorders

Patients can present with more than one vestibular disorder; hence, it is imperative to take a good history [28]. In addition, scales such as the Dizziness Handicap Inventory, [29] which evaluates the level of dizziness and the emotional, physical, and functional changes that accompanies dizziness, and the Vestibular Rehabilitation Benefit questionnaire [30], which includes an assessment of motion-induced dizziness, could be used to develop a robust diagnosis while ruling out other vestibular disorders. Other neuropsychological tests, including visuospatial tests such as intersecting pentagon copying, clock-face drawing, and block design tests have been reported to predict on-road driving behaviour [2]. Other psychological tests such as the Romberg test of standing balance on firm and compliant support surfaces can help determine the different systems involved in balance, such as the vestibular system, vision, and proprioception. This could in turn help determine the vestibular tone while driving, which dictates the feeling of vehicle veering, disorientation, nausea, and panic among drivers [2].

We also propose the following diagnostic criteria for primary MVDS in patients presenting with two or more symptoms of dizziness while driving on most days, such as:

- The vehicle might turn to one side
- Disorientation
- Pre-syncopal symptoms (sweating, cold extremities, palpitations)
- Postural adjustments *

- Symptoms are present exclusively while driving and aggravate during the following situations:
  - Higher speed (>80 km/h)
  - Bends and turns
  - Multi-lanes
  - Upward or downward slope
  - Looking down from bridges
  - Overtaking
  - Looking at other vehicles while driving
  - Closed tunnels
- The disorder usually begins insidiously without any identifiable precipitating event.
- Symptoms cause significant distress or functional impairment while driving.
- Symptoms are not better attributed to another disease or disorder **.

* Such as stiffness of body, holding the steering wheel tightly

** Patients devoid of de novo symptoms of visual vertigo or other vestibular disorders presenting while driving.

There is limited literature on the treatment of MVDS. The general principles of MVDS treatment are the exclusion of organic disease, treatment of general state anxiety/phobia, explanation of symptoms, and progressive desensitization. One approach to treatment involves grouping patients into two categories [31]:

(1) Patients with car-tilt illusions and previous vestibular disorders without panic disorders should receive vestibular rehabilitation with progressive desensitization.

(2) Patients with panic disorders without car-tilt illusions and vestibular disorders should receive selective serotonin reuptake inhibitors (SSRI), anxiolytics, and cognitive behavioral therapy (CBT).

In our study, we treated patients with various medications such as Amitriptyline, Venlafaxine, Pregabalin, Bisoprolol, Gabapentin, Pregabalin, and Magnesium with variable relief of symptoms. The natural history of MVDS ranges from complete recovery with or without treatment to chronic suffering without relief.

## 5. Limitations

As this was a two-center study, there was variation in the approach to history taking between the centers. Therefore, data concerning symptoms, etc., were not recorded for a few patients. The treatments were not uniform across all patients, and the of follow-up outcomes after treatment were not recorded in all the patients. Because this was a retrospective study, gaps in the data could not be addressed by following up with patients; therefore, a prospective, cross-sectional study could help to accurately identify patient characteristics.

## 6. Conclusions

MVDS is a distinct clinical entity that requires diagnostic consideration and treatment. The time to diagnose this condition can be reduced with widespread recognition. Most patients with MVDS have a history of migraines and non-specific video-oculographic abnormalities. We also propose a classification system, diagnostic criteria, and medical treatment options for MVDS.

## Figures and Tables

**Table 1:** Clinical characteristics of patients diagnosed with Motor Vestibular Disorientation Syndrome.

Clinical Characteristics		n (%)
History *	Migraine	15 (62.5)
	Hypertension	5 (20.8)
	Diabetes	3 (12.5)
	Hyperlipidemia	3 (12.5)
	Hypothyroidism	2 (8.3)
	No co-morbidity	9 (37.5)
Symptoms of MVDS *	Disorientation	19 (79.2)
	Sense of losing control	13 (54.2)
	Palpitations	10 (41.7)
	Vehicle turning to right	9 (37.5)
	Vehicle turning to left	8 (33.3)
	Sweating	6 (25)
	Stiffness of body	5 (20.8)
	Neck pain	3 (12.5)
	Blackout	2 (8.3)
	Cold extremities	1 (4.2)
Triggers *	Higher speed	16 (66.7)
	Multi-lanes	14 (58.3)
	Bends and turns	12 (50)
	Looking at other vehicles while driving or at signals	10 (41.7)
	Overtaking	7 (29.2)
	Upward slope	6 (25)
	Looking down from bridges	6 (25)
	Downward slope	5 (20.8)
	Closed tunnels	3 (12.5)
The side to which patients felt veering or turning of vehicle	Right side	9 (37.5)
	Left side	8 (33.3)
	No feeling	7 (29.2)
Time of the day when the symptoms were severe *	Anytime while driving	15 (62.5)
	Better in the morning and more in the evening	8(33.3)
	Morning	4 (16.7)
Other symptoms *	Headaches	15 (62.5)
	Fear/dizziness in open and or crowded places, supermarkets, malls, airport	5 (22.7)
	Phonophobia	5 (22.7)
	Blurred vision	4 (18.2)
	Photophobia	2 (9.1)
	None	4 (18.2)
Symptoms of motion sickness (Current or past) (n = 12) #	Nausea/Vomiting	5 (41.7)
	Drowsiness	4 (33.3)
	Headache	4 (33.3)
	Fatigue	3 (25)
	Giddiness/Imbalance	1 (8.3)
	Irritability	1 (8.3)
	Yawning	1 (8.3)

* Patients chose more than one option # Out of 12 patients who reported motion sickness while traveling as a passenger. MVDS- Motorist vestibular disorientation syndrome

**Table 2:** Video-nystagmography recordings of 17 patients.

Parameters		n (%)
Horizontal Saccades	Normal	17 (100)
Vertical Saccades	Normal	15 (88.2)
	Hypometric	2 (11.8)
Horizontal Smooth Pursuit	Normal	13 (76.5)
	Saccadic	4 (23.5)
Vertical Smooth Pursuit	Normal	1 (5.9)
	Saccadic	16 (94.1)
Spontaneous nystagmus (SPN) in dark (without fixation)	No	14 (82.4)
	Up beating nystagmus	3 (17.6)
Head-Shaking nystagmus	Absent	14 (82.4)
	Present	3 (17.6)
Hyperventilation induced nystagmus	Absent	14 (82.4)
	Present	3 (17.6)

**Table 3:** Gaze without fixation.

Gaze without Fixation	Nystagmus (n)	No Nystagmus (n)
Centre (n = 15)	3	12
Left (n = 15)	2	13
Up (n = 16)	2	14
Right (n = 16)	2	14
Down (n = 15)	2	13

**Table 4:** Management methods used in patients and subsequent improvements.

Patient No.	Medications Used in Different Patients	Subjective Report of Improvement
1.	Amitriptyline, Bisoprolol 2.5, Magnesium (400 mg)	40% better in Amitriptyline, near complete on the addition of Bisoprolol 2.5, Mg 400 mg
2.	Amitriptyline	Didn’t drive for initial 2–3 months, Improved after treatment
3.	Amitriptyline, Bisoprolol	70% better
4.	Carbamazepine (associated epilepsy)	80% better
5.	Amitriptyline	No improvement
6.	Amitriptyline, Bisoprolol, Magnesium, Desvenlafaxine	No improvement with Amitriptyline, 30–40% with Bisoprolol and Mg, 80% with Desvenlafaxine
7.	Topiramate	Follow-up not available
8.	Amitriptyline	Follow-up not available
9.	Eslicarbazepine, Gabapentin 300 (vascular loop on MRI)	No improvement, Side-effects with Eslicarbazepine 800 mg, 70–80% improvement after Gabapentin
10.	Amitriptyline	80% improvement
11.	Pregabalin	75% improvement
12.	Carbamazepine, Pregabalin, Amitriptyline, Magnesium, Gabapentin 300 (vascular loop on MRI)	No improvement with Carbamazepine and Pregabalin, 50% on Amitriptyline and Mg, 80–90% on Gabapentin
13.	No treatment	Felt better without any treatment
14.	No treatment	Didn’t follow-up
15.	Venlafaxine	25–30% after Venlafaxine
16.	Magnesium, Concor	NA

Abbreviations: Mg—Magnesium.

## Data Availability

Data is available from the corresponding author upon reasonable request.
